# Co-modification of corn flour by *Lactobacillus HR* and transglutaminase reduces glycemic index and enhances functional properties

**DOI:** 10.3389/fnut.2026.1737314

**Published:** 2026-02-04

**Authors:** Xingda Xu, Yifan Guo, Hongdi Sun, Yutong Zhang, Wanting Yang, Hua Zheng, Lei Xu, Yan Wang

**Affiliations:** 1 College of Food and Biological Engineering, Qiqihar University, Qiqihar, China; 2 Engineering Research Center of Plant Food Processing Technology, Ministry of Education, Qiqihar, China; 3 Crop Resources Institute, Heilongjiang Academy of Agricultural Sciences, Harbin, China; 4 Tonghua Central Hospital, Tonghua, China

**Keywords:** bacteria-enzyme co-midification, cereal processing, corn flour, low glycemic index, optimization

## Abstract

**Introduction:**

The complex protein structure, high glycemic index (GI), coarse taste and poor formability of corn significantly hinder its deep processing performance and represent key technical bottlenecks in the industrialization of corn-based food products.

**Methods:**

In this study, co-modification with optimized lactic acid bacteria (Lactobacillus HR) and transglutaminase (TGase) effectively converted corn flour into a low-glycemic index (GI) product, primarily due to flour’s high starch content.

**Results:**

The results revealed that fermentation under the optimum circumstances (36.83 °C, fermentation time of 49.25 h, inoculum level of 1.03 × 108 CFU/mL, and enzyme dosage of 2.58‰) produced flour with significant nutritional improvements. Co-modification yielded the lowest GI (48.87) and enhanced the nutritional profile, with increased soluble protein content (5.96  mg/g) and resistant starch levels (51.19%). FTIR analysis revealed an increase in the (1047/1022) cm^−1^ absorbance ratio, suggesting a higher degree of starch molecular order and consequently, enhanced resistance to enzymatic digestion. Compared with untreated corn flour, the treated samples showed a 10.3% decrease in water-holding capacity and a 29.7% increase in gel strength.

**Conclusion:**

The co- modification of Lactobacillus HR and TGase caused a more compact internal structure, which further reduced the hydrolyzability of corn starch. Based on the above results, this study indicates that the integrated biological treatment is a feasible method for improving the nutritional quality and functional properties of cereal flours.

## 1 Introduction

Corn (*Zea mays*), an annual grass of the family poaceae plant, is a globally vital food crop due to high yield and extensive cultivation. China ranked second globally in cultivation area (2023) (1). In addition to its agricultural importance, corn is also rich in dietary fiber (cellulose), amino acids (including glutamic acid), unsaturated fatty acids, protein, starch, vitamins A, B1, B2, and E, carotenoids, and minerals (calcium, phosphorus, iron), and it serves as an important metabolic energy source (2, 3). Corn flour has high water retention capacity/oil-absorption capacity (4). As a staple food, corn offers health benefits (5), including provitamin A carotenoids (such as *β*-carotene) which are converted into vitamin A—a nutrient which is essential for vision and immune function (6), antioxidants (7) (e.g., reduced glutathione, GSH) that support chemotherapy and scavenge free radicals, and lignin which boosts immunity by stimulating macrophages (8). Owing to its unique structure, corn lacks gluten-forming proteins (9, 10), which makes the corn flour difficult to form cohesive dough; such dough exhibits cracking tendencies, inferior viscoelasticity and chewiness, and a rough texture, greatly constraining its processing ability and preventing its direct use as a raw material (11, 12). Therefore, corn has been used throughout history for energy production, chemical raw material, and as animal feed. However, intensive processing by these departments often results in a significant waste of food resources and a loss of their inherent nutritional value.

With the rising consumer demand for food quality, texture, and functionality, improving the processing performance of corn flour has become crucial, and technologies aimed at enhancing corn flour quality have gradually emerged as a focus of attention in the industry (13). Studies have illustrated that physical (14), chemical (15), and biological modification technologies can effectively increase the specific surface area and water-binding capacity of corn flour, making it more amenable to processing and shaping. In this context, certain enzyme technologies for the structural and functional modification of cereal flour are employed, including pullulanase (16), glucanotransferase (17), and amylase (18), to alter the structural characteristics of starch and its functional properties such as viscosity and water absorption. These technologies can significantly improve corn flour’s processing performance, quality, and nutritional composition. Owing to these changes, the flour can be applied in various applications in the food industry. *Lactobacillus HR* is renowned for their highly efficient secretion of starch hydrolase, which is beneficial for altering the digestibility of starch (19). In addition, TGase catalyzes the cross-linking between protein molecules, thereby creating an enhanced cross-linked protein network in the corn flour matrix. The combination of *Lactobacillus HR* fermentation and TGase treatment is a promising comprehensive modification and synergistic strategy for corn flour, aiming to simultaneously enhance the processing performance of corn flour and reduce its impact on blood sugar. For instance, fermented indica rice with *Lactobacillus plantarum* significantly increased the contents of resistant starch (RS) and slowly digestible starch (SDS), enhanced the short-range ordered structure and relative crystallinity of the starch, and thereby reduced rice digestibility and its glycemic index (GI) (20). Such modifications yield functional foods with health benefits, such as lowering pre-existing glycemic levels (21).

The concept of the GI, which measures the impact of carbohydrate-containing foods on postprandial blood-glucose levels, was first introduced by David Jenkins et al. in the early 1980s (22). It compares the blood-glucose response level over 2 h after consuming 50 g of carbohydrates from a test food to equivalent amount of glucose or wheat bread (23). Foods are classified as high (GI > 70), medium (GI 55–70), and low (GI < 55), based on their GI values (24). Research has indicated that consuming low-GI foods over an extended period of time can reduce blood glucose, blood lipids, and obesity (25). According to the studies, switching from high-GI to low-GI foods has minimal impact on blood sugar levels during the intermediate stages of the disease, although there is still some statistical significance, predominantly in diabetes management (26–28). The enzymatic modification can form resistance starch (RS), which contributes to glycemic control in different starch sources, particularly corn (29).

Based on the above background, we proposed a synergistic modification strategy, combining *Lactobacillus HR* fermentation with transglutaminase (TGase) treatment as a novel approach to simultaneously address the challenges related to corn flour processing and nutrition. Accordingly, this study aimed to explore the efficacy and mechanism of this co-modification in reducing the glycemic index (GI) of corn flour while maintaining or improving its functional properties. For this purpose, the response surface method is adopted to optimize the fermentation process. The effects of co-modification on water retention performance and gel performance were systematically evaluated. The microstructure evolution and molecular-level changes were observed and analyzed using scanning electron microscopy (SEM) and Fourier transform infrared spectroscopy (FTIR). Nutritional components and amino-acid composition were quantified throughout the modification process. Through this multi-scale analysis, we aim to comprehensively elaborate on the potential by which *Lactobacillus HR* and TGase jointly regulate starch digestibility and blood glucose response, thereby providing a theoretical basis for the development of low-GI corn-based functional foods.

## 2 Materials and methods

### 2.1 Material

The commercial corn flour was produced from a common yellow corn variety (*Zea mays* L.) purchased from China Neihe Tianfeng Flour Co., Ltd., and obtained through grinding and sieving. *α*-Amylase (4,000 U/g), neutral protease (100 U/mg), cellulase (400 U/mg), compound protease (120 U/mg), papain (800 U/mg), and transglutaminase (TG, 200 U/g) were purchased from Nanning Dongheng Huadao Biotechnology Co., Ltd. (Nanning, China). *Lactobacillus HR* is a research strain preserved by the Culture Collection Center of the College of Food and Biological Engineering, Qiqihar University (Qiqihar, Heilongjiang, China). All other chemicals and reagents were purchased from Tianjin Kaitong Chemical Reagent Co., Ltd. (Tianjin, China) and were of analytical grade.

### 2.2 Methods

#### 2.2.1 Determination of soluble protein content

The soluble protein content was determined using the Folin–Ciocalteu method, adapted from Uraipong et al. (30). A 250 μg/mL bovine serum albumin (BSA) standard solution was prepared. Aliquots of 0.0, 0.1, 0.2, 0.4, 0.6, 0.8, and 1.0 mL of the standard were pipetted into test tubes and diluted to 1 mL with distilled water. Subsequently, 5 mL of reagent A (containing 4% Na_2_CO_3_, 0.2 mol/L NaOH, 1% CuSO_4_, and 2% potassium sodium tartrate) was added to each tube. After mixing, the tubes were incubated in a water bath at 20–25 °C for 10 min. Then, 0.5 mL of reagent B (commercial Folin–Ciocalteu reagent) was added, and the mixture was incubated at 30 °C for 30 min to allow color development. Absorbance was measured at 640 nm. A standard curve was constructed by plotting protein concentration against mean absorbance. For sample analysis, the protein was extracted from the corn flour and was appropriately diluted to fall within the linear range of the standard curve. Color development and measurement followed the same procedure, and soluble protein content was calculated from the standard curve.

#### 2.2.2 Determination of starch digestibility

The determination of starch digestibility was carried out according to the reported method with minor modifications (31). Approximately 100 mg of the ground sample was added to a 50 mL cap-filled test tube, followed by the addition of 9 mL of acetic acid–sodium acetate buffer solution (0.2 M, pH 5.8). The mixture was shaken for 30 min in a water bath at 37 °C. Subsequently, 1 mL of the mixed enzyme solution was added and thoroughly mixed. The enzyme solution contained *α*-amylase (250 U/mL), amyloglucosidase (3,000 U/mL), and trypsin (1 mg/mL) at a volume ratio of 7.9:0.5:1.6, simulating gastrointestinal digestion conditions. The mixture was incubated in a water bath at 37 °C under constant temperature. At digestion times of 0, 10, 20, 30, 45, 60, 90, 120, and 180 min, 0.2 mL of the digestion solution was taken from the sample and added to a centrifuge tube containing 1.8 mL of anhydrous ethanol. The mixture was shaken and centrifuged, and the supernatant was collected. The glucose content in the supernatant was quantified using the 3,5-dinitrosalicylic acid (DNS) method by measuring absorbance at 550 nm against a glucose standard curve.

#### 2.2.3 Calculation method for GI

The glucose standard curve was used as a reference, and the area under the *in vitro* starch-digestion curve (AUC) from 0 to 180 min was calculated using Origin 2018 software (32). The starch hydrolysis rate is calculated according to the Equation 1, the HI (Hydrolysis Index) is calculated according to Equation 2. After obtaining the hydrolysis index, the GI (Glycemic Index) is calculated according to Equation 3.


(1) $$\text{Starch hydrolysis}\;(\%)=\frac{G\times 0.9\times D\times V}{W}\times 100$$

(2) $$\mathrm{HI}\;(\%)=\frac{{\mathrm{AUC}}_{\text{sample}}}{{\mathrm{AUC}}_{\text{standard sample}\;(\text{white bread})}}\times 100$$

(3) GI=39.71+(0.549×HI)

where G is the amount of glucose released (mg/mL), D is the dilution factor, V is the total volume of the reaction mixture (mL), and W is the dry weight of the corn flour sample (mg).

AUC_sample_ is the area under the *in vitro* digestion curve of starch for the sample from 0 to 180 min, and AUC_standard sample (white bread)_ is the area under the in vitro digestion curve of starch for the standard sample, white bread, from 0 to 180 min.

#### 2.2.4 Resistant starch calculation method

The resistant starch content was calculated using the following the Equation 4:


(4) $$\mathrm{RS}(g/100g\;\text{starch})=\frac{\mathrm{TS}-(G_{120}-G_{0})\times 0.9}{\mathrm{TS}}\times 100$$

where G_120_ is the glucose content in the supernatant at 120 min digestion, G_0_ is the glucose content in the supernatant at 0 min digestion, and TS is the total starch content.

#### 2.2.5 Corn flour fermentation optimization

Solid-state fermentation was performed in a constant temperature incubator (Model: PYX-DHS, Shanghai Yuejin Medical Instrument Factory, China). Corn flour was used as a solid fermentation medium, and the *Lactobacillus HR* culture was inoculated to initiate the fermentation process. The effects of several factors were investigated, including inoculation rate (3 × 10^7^–1.5 × 10^8^ CFU/mL), moisture content of the material (30–50% v/m), fermentation temperature (33 °C-41 °C), and fermentation time (24–72 h). During this fermentation process, the GI value, resistant starch content, and soluble protein content were evaluated.

#### 2.2.6 Enzyme screening

Based on the determined corn flour fermentation process, *α*-amylase, neutral protease, cellulase, composite protease, papain, and TGase were, respectively, added at a dose of 90 U/g of corn flour. The mixture was then incubated at 37 °C for 180 min, after which the GI value was determined.

#### 2.2.7 Bacterial-enzyme synergistic treatment of corn flour

##### 2.2.7.1 Single-factor fermentation

Corn flour was used as a solid fermentation medium, and the *Lactobacillus HR* culture was inoculated for solid fermentation. The effects of inoculation amount (3 × 10^7^–1.5 × 10^8^ CFU/mL), enzyme addition amount (1–5‰, w/w), fermentation temperature (33–41 °C), and fermentation time (24–72 h) on GI value, resistant starch content, and soluble protein content were systematically investigated.

##### 2.2.7.2 Optimization of fermentation process by box–Behnken test

Based on single-factor experiments, the fermentation process was optimized using response surface methodology (RSM). A Box–Behnken design (BBD) was generated using Design Expert^®^ (version 8.0, Stat-Ease, United States) to evaluate both the individual and interactive effects of four key factors: TGase addition, *Lactobacillus HR* inoculation level, fermentation time, and fermentation temperature. Each factor was studied at three coded levels (−1, 0, and +1). The GI value of the co-modified corn flour was selected as the response variable for model fitting and for identifying the optimal fermentation conditions.

#### 2.2.8 Determination of the water-holding capacity of four corn flours

For each of the four corn-flour samples, 2.00 g was accurately weighed and mixed with 10.0 mL of distilled water. After thorough homogenization, the mixture was centrifuged at 4,500 rpm for 12 min. The supernatant was then decanted, and its mass was recorded. The following Equation 5 was used to determine the water retention:


(5) $$\text{Water retention}\;(g/g)=\frac{10-W}{2\;}$$

where W is the mass of the supernatant.

#### 2.2.9 Determination of gel strength of four corn flours

For each corn-flour sample, 2.00 g was accurately weighed and mixed with 10.0 mL of distilled water. After thorough homogenization, the mixture was boiled for 10 min, and the supernatant was discarded. The following Equation 6 was employed to determine the gel strength:


(6) $$\mathrm{Gel}\;\text{strength}\;(g/g)=\frac{W-W_{0}}{2}$$

where W is the total mass of the test tube and residue after removal of the supernatant, and W_0_ is the mass of the test tube.

##### 2.2.9.1 Determination of nutritional components

The determination of the basic nutritional components of corn flour was conducted according to the methods of the Association of Official Analytical Chemists (AOAC) (33). Among them, the moisture content is determined by the direct drying method (AOAC 925.10). The ash content was determined by the calcination method (AOAC 942.05). The protein content was analyzed by the Kjeldahl nitrogen determination method (AOAC 928.08). The fat content was quantified by Soxhlet extraction (AOAC 920.39). The starch content was assessed through enzymatic hydrolysis (AOAC 996.11). The content of crude fiber was measured by acid–base digestion (AOAC 978.101). The total sugar content was determined by the film reagent titration method (AOAC 923.09).

#### 2.2.10 Surface microstructure

An appropriate amount of corn flour was evenly dispersed and stuck to the sample stage using double-sided conductive tape, ensuring that the cross-sectional surface faced upward.

The prepared samples were mounted on the stage of a SEM, and their surface morphology was observed and imaged at magnifications of 500×, 1,000×, 2,000×, and 4,000 × under an accelerating voltage of 20 kV (34).

#### 2.2.11 Fourier transform infrared spectroscopy

FT-IR analysis was conducted to investigate the arrangement of starch chains at the particle surface. The penetration depth of the IR beam was set to 2 μm. For KBr pellet preparation, 2 mg of the sample was accurately weighed and mixed with 0.200 g of potassium bromide (KBr), then the sample was ground thoroughly to a homogeneous powder and pressed into a transparent pellet using a pellet die. The spectra were recorded in the range of 400–4,000 cm^−1^ to obtain the infrared spectrum of the sample (35, 36).

#### 2.2.12 Determination of amino acid composition

Amino acid analysis was conducted via acid hydrolysis following the GB/T 14965–1994 standard.

#### 2.2.13 Statistical analysis

All the experiments were conducted in triplicate. Data were processed and analyzed using SPSS 19.0 (SPSS Inc., Chicago, IL, United States) and are expressed as mean ± standard deviation. Statistical significance was assessed using one-way analysis of variance (ANOVA), with significance defined at *p* < 0.05. Figures were generated using Origin 2024 (OriginLab Corporation, Northampton, MA, United States).

## 3 Results

### 3.1 Study on the technology of fermented corn flour

**Figure 1:**
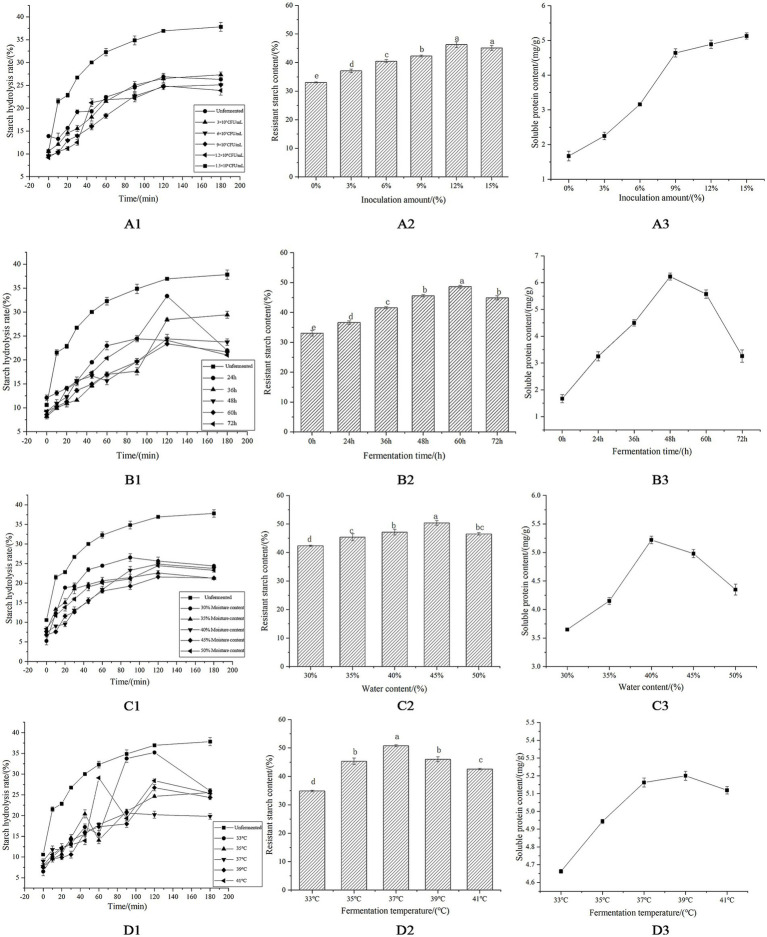
Processing and optimization of corn flour by microbial treatment. **(A1)** Effect of inoculation amount on the hydrolysis rate of starch. **(A2)** Effect of inoculation amount on resistant starch content. **(A3)** Effect of inoculation amount on soluble protein content. **(B1)** Effect of fermentation time on the hydrolysis rate of starch. **(B2)** Effect of fermentation time on resistant starch content. **(B3)** Effect of fermentation time on soluble protein content. **(C1)** Effect of water content on the hydrolysis rate of starch. **(C2)** Effect of water content on resistant starch content. **(C3)** Effect of water content on soluble protein content. **(D1)** Effect of fermentation temperature on the hydrolysis rate of starch. **(D2)** Effect of fermentation temperature on resistant starch content. **(D3)** Effect of fermentation temperature on soluble protein content.

Regarding inoculum size (3 × 10^7^–1.5 × 10^8^ CFU/mL), an inoculum of 1.2 × 10^8^ CFU/mL led to a substantial reduction in GI (51.8) (Figure 1A1), and a significant increase in RS content (*p* < 0.05), attributed to the activity of *Lactobacillus HR* (Table 1) while the soluble protein content also showed a corresponding change (Figure 1A3). In contrast, a higher inoculum of 1.5 × 10^8^ CFU/mL resulted in overfermentation (Figure 1A2). RS is metabolized through fermentation, which not only leads to a decrease in its content but also produces an unpleasant flavor. Therefore, the inoculum of 1.2 × 10^8^ CFU/mL was more favorable for reducing the GI.

**Table 1:** Effects of varying inoculation amount, fermentation time, water content, and temperature on the hydrolysis index (HI) and the glycemic index (GI).

Parameter (s)	Level	HI (mean ± SD)	GI (mean ± SD)
Inoculation amount	Unfermented	36.93 ± 1.17 ^a^	59.98 ± 1.44 ^a^
	3 × 10^7^ CFU/mL	29.81 ± 0.56 ^b^	56.07 ± 0.78 ^b^
	6 × 10^7^ CFU/mL	27.46 ± 0.91 ^c^	54.78 ± 1.19 ^b^
	9 × 10^7^ CFU/mL	23.90 ± 0.69 ^d^	52.83 ± 0.41 ^c^
	1.2 × 10^8^ CFU/mL	21.97 ± 0.54 ^e^	51.77 ± 0.68 ^c^
	1.5 × 10^8^ CFU/mL	23.65 ± 1.12 ^d^	52.69 ± 1.20 ^c^
Fermentation time	Unfermented	36.93 ± 1.17 ^a^	59.98 ± 1.44 ^a^
	24 h	27.78 ± 0.96 ^b^	54.96 ± 0.78 ^b^
	36 h	23.36 ± 0.25 ^c^	52.53 ± 0.23 ^c^
	48 h	22.02 ± 0.59 ^de^	51.79 ± 0.63 ^c^
	60 h	21.07 ± 0.89 ^e^	51.27 ± 0.21 ^d^
	72 h	23.08 ± 1.17 ^cd^	52.38 ± 1.09 ^c^
Water content	Unfermented	36.93 ± 1.17 ^a^	59.98 ± 1.44 ^a^
	30%	26.38 ± 0.09 ^b^	54.19 ± 0.18 ^b^
	35%	23.43 ± 1.25 ^c^	52.57 ± 0.93 ^c^
	40%	22.13 ± 0.99 ^c^	51.85 ± 0.83 ^cd^
	45%	20.36 ± 0.77 ^d^	50.89 ± 0.71 ^d^
	50%	22.34 ± 0.17 ^c^	51.97 ± 0.09 ^cd^
Temperature	Unfermented	36.93 ± 1.17 ^a^	59.98 ± 1.44 ^a^
	33 °C	27.88 ± 0.29 ^b^	55.02 ± 0.15 ^b^
	35 °C	22.51 ± 0.95 ^d^	52.06 ± 0.83 ^d^
	37 °C	20.35 ± 0.27 ^e^	50.88 ± 0.43 ^e^
	39 °C	21.96 ± 0.87 ^d^	51.77 ± 0.51 ^d^
	41°C	24.79 ± 0.12 ^c^	53.32 ± 0.05 ^c^

Data are expressed as mean ± standard deviation (SD). Lower-case letters (a, b, c, d, e) denote statistically significant differences (*p* < 0.05) among treatments within each parameter.

Under the above-mentioned inoculum conditions, drastic starch breakdown occurred at 60 h (Figure 1B1), reducing the GI to approximate value of 51.3 due to indigestible components (Table 1). RS content and soluble protein levels peaked between 48 and 60 h, whereas fermentation beyond 60 h led to a decline in *Lactobacillus HR* populations, and the RS and soluble proteins might be further hydrolyzed (Figures 1B2,B3).

Moisture content analysis showed that 45% moisture achieved the lowest GI and highest RS levels (Table 1 and Figure 1C2) with “Moisture content analysis showed that starch hydrolysis was influenced by different moisture levels (Figure 1C1). A moisture content of 45% achieved the lowest GI and highest RS levels (Table 1 and Figure 1C2).

Temperature optimization studies found that 33–37 °C supported microbial metabolism and enzymatic activity, decreasing GI, and increasing RS content (Figure 1D1). Temperatures above 37 °C had negative effects on the fermentation process, thereby increasing GI and decreasing RS formation (Table 1 and Figure 1D2). Soluble protein production peaked at about 39 °C before a decline (Figure 1D3).

Comprehensive analysis determined the optimal fermentation parameters as follows: 1.2 × 10^8^ CFU/mL inoculum, 60-h fermentation, 45% moisture, and 37 °C temperature. These conditions balanced bacterial activity, starch modification, and enzyme stability to maximize RS and minimize GI.

### 3.2 Enzyme screening

**Figure 2:**
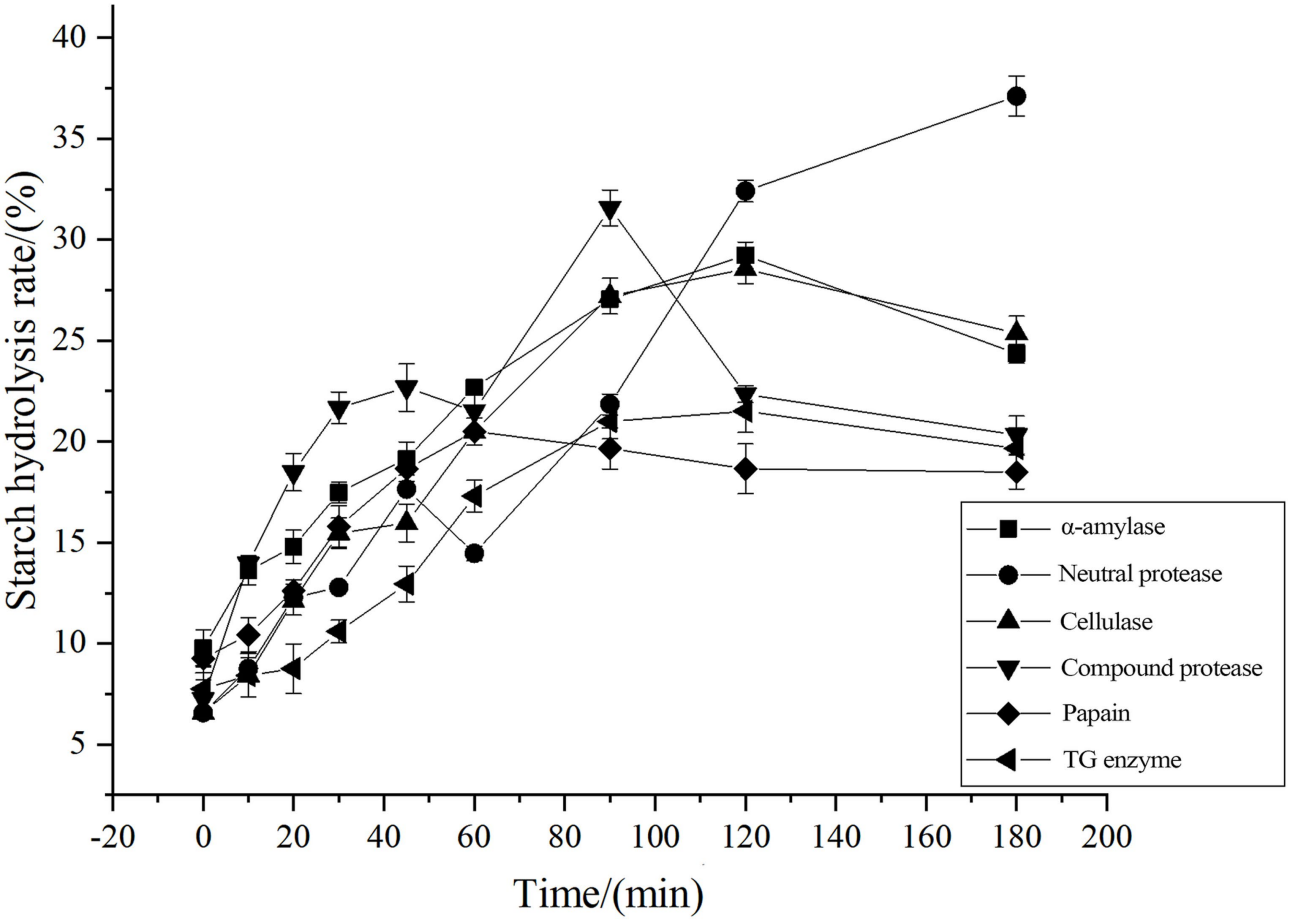
Comparison of starch hydrolysis rates under the action of different enzymes.

Figure 2 shows the effects of six different enzymes (*α* -amylase, neutral protease, cellulase, complex protease, papain, and TG enzyme) on the starch hydrolysis rate within a reaction time of 180 min. α -amylase exhibits significant hydrolytic activity, with its reaction curve rising rapidly, achieving a starch hydrolysis rate of approximately 35% within 60 min. In contrast, the starch hydrolysis rates catalyzed by the other five enzymes remained at an extremely low level (below 5%) throughout the entire reaction process, and their reaction curves were gentle with no obvious upward trend. Neutral protease, cellulase, complex protease, papain, and TGase mainly target cereal proteins. Moderate proteolysis might improve the texture of food, while slowing down the enzymatic digestion rate of starch in the food matrix through synergistic means such as physical embedding, structural reorganization, and cross-linking with dietary fiber.

Further enzyme screening is carried out based on an optimal fermentation process for selecting enzymes that have a positive impact on GI value when combined with *Lactobacillus HR* (Table 2). Different enzyme treatments have significant effects on the hydrolysis index (HI) and GI of corn flour. *α* -amylase and neutral protease significantly increased HI and GI values, indicating that they promote starch digestion. Cellulase and complex protease also cause a slight increase in the GI value. It is worth noting that papain and TGase significantly reduced HI and GI values. The mechanism may lie in their action on protein components, delaying digestion by altering the state of starch coating or forming a protein network. Moreover, although there is no significant difference between papain and TGase at the overall level (*p* > 0.05), TGase has a relatively low HI and GI index. This indicates that TGases are enzymes with the potential to reduce the GI of corn flour. However, during the fermentation of corn flour, the pH value decreased (37), which likely inhibited the activity of neutral protease and composite protease. This also indicates that these two enzymes are not suitable for co-fermentation with acid-producing bacterial strains.

**Table 2:** Effect of enzyme types on hydrolysis index (HI) and glycemic index (GI) of corn flour.

Enzyme screening	Condition	HI (mean ± SD)	GI (mean ± SD)
Fermentation without enzyme	0 μ/g	20.35 ± 0.27 ^c^	50.88 ± 0.43 ^c^
α-Amylase	90 μ/g	23.36 ± 0.17 ^a^	52.54 ± 0.44 ^a^
Neutral protease	90 μ/g	23.44 ± 0.79 ^a^	52.57 ± 0.62 ^a^
Cellulase	90 μ/g	22.18 ± 0.25 ^b^	51.89 ± 0.93 ^b^
Compound protease	90 μ/g	22.32 ± 0.77 ^b^	51.96 ± 0.83 ^b^
Papain	90 μ/g	17.77 ± 0.56 ^d^	49.46 ± 0.35 ^d^
TGase enzyme	90 μ/g	17.30 ± 0.57 ^d^	49.20 ± 0.79 ^d^

Data are expressed as mean ± standard deviation (SD). Lower-case letters (a, b, c, d) denote statistically significant differences (*p* < 0.05) among treatments within each parameter.

### 3.3 Process optimization of bacterial-enzyme synergistic treatment of corn flour

**Figure 3:**
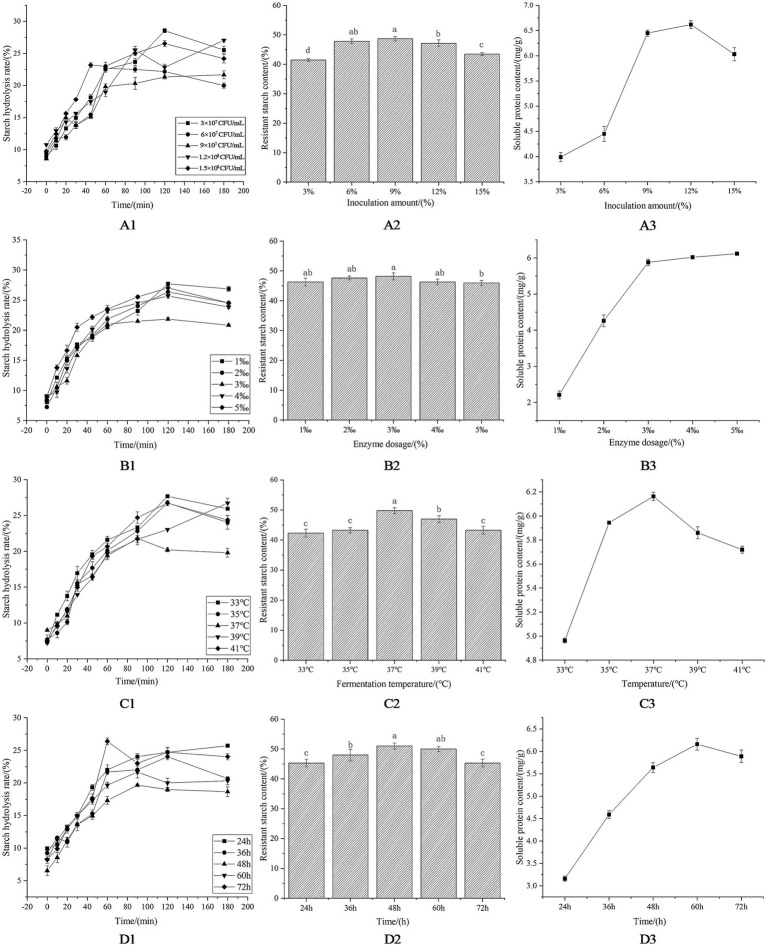
Synergistic microbial-enzymatic treatment and process optimization of corn flour. **(A1)** Effect of inoculation amount on the hydrolysis rate of starch. **(A2)** Effect of inoculation amount on resistant starch content. **(A3)** Effect of inoculation amount on soluble protein content. **(B1)** Starch hydrolysis rate of corn flour with different enzyme dosages. **(B2)** Resistant starch content of corn flour with different enzyme dosages. **(B3)** Soluble protein content of corn flour with different enzyme dosages. **(C1)** Effect of temperature on the hydrolysis rate of starch. **(C2)** Effect of temperature on resistant starch content. **(C3)** Effect of temperature on soluble protein content. **(D1)** Effect of time on the hydrolysis rate of starch. **(D2)** Effect of time on resistant starch content. **(D3)** Effect of time on soluble protein content.

Regarding the inoculum amount, the glycemic index (GI) of corn flour initially decreased and subsequently increased with varying inoculum levels (Table 3). In contrast, the content of RS and soluble protein contents showed an inverse trend (Figures 3A2,A3). An intermediate inoculum of 9 × 10^7^ CFU/mL was identified as optimal for maximizing microorganism-enzyme synergy and was selected for subsequent experiments. Exceeding this threshold led to nutrient insufficiency per cell, undermining synergy and reducing fermentation efficiency.

**Table 3:** Effects of fermentation and enzymatic hydrolysis parameters on HI and GI of corn flour.

Factor	Level	HI (mean ± SD)	GI (mean ± SD)
Inoculation amount (%)	3 × 10^7^ CFU/mL	22.27 ± 0.28 ^a^	51.94 ± 0.74 ^a^
	6 × 10^7^ CFU/mL	19.28 ± 0.53 ^c^	50.29 ± 0.96 ^b c^
	9 × 10^7^ CFU/mL	18.73 ± 0.36 ^c^	49.99 ± 0.03 ^c^
	1.2 × 10^8^ CFU/mL	21.20 ± 0.21 ^b^	51.34 ± 0.66 ^a b^
	1.5 × 10^8^ CFU/mL	22.71 ± 0.13 ^a^	52.17 ± 0.21 ^a^
Enzyme dosage (‰)	1‰ (30 μ/g)	25.70 ± 0.71 ^a^	53.82 ± 0.68 ^a^
	2‰ (75 μ/g)	20.08 ± 0.35 ^c^	50.73 ± 1.14 ^c^
	3‰ (150 μ/g)	18.31 ± 0.33 ^d^	49.76 ± 0.39 ^d^
	4‰ (225 μ/g)	20.45 ± 0.09 ^c^	50.94 ± 0.76 ^c^
	5‰ (300 μ/g)	21.95 ± 0.21 ^b^	51.76 ± 0.53 ^b^
Temperature (°C)	33 °C	22.23 ± 0.11 ^a^	51.91 ± 0.17 ^a^
	35°C	20.82 ± 0.89 ^b c^	51.14 ± 0.42 ^b c^
	37 °C	18.27 ± 0.21 ^d^	49.73 ± 0.31 ^d^
	39 °C	20.01 ± 0.77 ^c^	50.69 ± 0.48 ^c^
	41°C	21.49 ± 0.13 ^a b^	51.51 ± 0.05 ^a b^
Time (h)	24 h	21.45 ± 0.12 ^a^	51.48 ± 0.08 ^a^
	36 h	19.56 ± 0.89 ^b^	50.45 ± 0.77 ^a b^
	48 h	16.77 ± 0.69 ^c^	48.92 ± 0.91 ^c^
	60 h	18.53 ± 0.77 ^b^	49.88 ± 0.93 ^b c^
	72 h	21.32 ± 0.67 ^a^	51.41 ± 0.34 ^a^

Data represent mean ± SD of triplicate measurements. Superscript letters (a, b) denote statistically significant differences (*p* < 0.05) within each parameter.

With respect to enzyme dosage, the observed trend was analogous to inoculum effects: GI initially decreased and then increased with rising enzyme concentrations, while RS contents first increased and then declined. In contrast, soluble protein contents rapidly increased initially before stabilizing at higher doses (Figures 3B2,B3 and Table 3). Excessive enzyme addition beyond saturation caused substrate saturation, which interfered with microbial proliferation and metabolism, disrupted the enzyme–microbe balance, and impaired product quality and yield.

With respect to temperature variations, GI decreased initially and then increased as temperature rose, while RS and soluble protein concentrations showed opposite trends (Figures 3C2,C3 and Table 3). Temperatures exceeding the physiological optimum reduce microbial viability and enzymatic activity and, in some cases, cause complete thermal inactivation, alter metabolic by-product profiles, and affect starch digestibility (Figure 3C1) (38). A comprehensive analysis determined that 37 °C is the optimal temperature for maintaining biological activity and product quality.

As for the time course, GI first increased and then decreased over time, while RS and soluble protein contents showed opposite trends (Figures 3D2,D3 and Table 3). The 48-h mark was identified as the optimal processing window, achieving minimum GI, maximum RS accumulation, and high soluble protein levels simultaneously. This duration is 12 h shorter than conventional single-strain fermentation, establishing it as the most efficient co-processing duration that balances product quality and economic feasibility.

### 3.4 Box–Behnken test to optimize the fermentation process

The optimal results of enzyme dosage, inoculum level, fermentation time, and fermentation temperature obtained from single-factor experiments were used to establish the level ranges for each factor in subsequent optimization. The fermentation process was optimized with GI value as the response variable. The results are shown in Tables 4, 5.

**Table 4:** Table of Box–Behnken experimental optimization results.

Serial number	GI value				
	A enzyme dosage (‰)	B Inoculation amount (%)	C time (h)	D Temperature (°C)	GI value
1	4	9	48	39	50.78
2	3	9	36	35	50.59
3	3	6	48	39	50.26
4	3	6	36	37	50.28
5	3	9	48	37	48.93
6	3	12	48	35	49.05
7	2	9	36	37	50.72
8	3	9	48	37	48.66
9	3	9	60	35	49.28
10	2	6	48	37	50.82
11	2	9	48	35	50.51
12	3	9	48	37	48.69
13	3	9	36	39	50.33
14	3	12	60	37	49.35
15	4	9	60	37	50.59
16	3	6	60	37	50.29
17	4	9	48	35	49.95
18	3	12	48	39	49.09
19	2	9	60	37	50.76
20	4	12	48	37	49.71
21	3	9	48	37	48.56
22	3	12	36	37	49.41
23	4	9	36	37	50.74
24	4	6	48	37	50.79
25	3	9	48	37	48.76
26	2	12	48	37	50.48
27	2	9	48	39	50.79
28	3	9	60	39	49.96
29	3	6	48	35	49.34

**Table 5:** Regression model analysis results table.

Source	Sum of squares	Degree of freedom	Equal square	*F*-value	*p*-value	Significance
Model	16.57	14	1.18	22.12	<0.0001	**
A	0.1850	1	0.1850	3.46	0.0841	
B	1.81	1	1.81	33.83	<0.0001	**
C	0.2821	1	0.2821	5.27	0.0376	*
D	0.5167	1	0.5167	9.66	0.0077	*
AB	0.1482	1	0.1482	2.77	0.1182	
AC	0.0090	1	0.0090	0.1687	0.6875	
AD	0.0756	1	0.0756	1.41	0.2542	
BC	0.0012	1	0.0012	0.0229	0.8819	
BD	0.1936	1	0.1936	3.62	0.0779	
CD	0.2209	1	0.2209	4.13	0.0616	
A2	11.14	1	11.14	208.18	<0.0001	**
B2	0.7454	1	0.7454	13.94	0.0022	*
C2	3.86	1	3.86	72.18	<0.0001	**
D2	1.47	1	1.47	27.39	0.0001	*
Residual	0.7489	14	0.0535			
Undrafted item	0.6886	10	0.0689	4.57	0.0781	
Pure error	0.0603	4	0.0151			
Total error	17.32	28				
*R*^2^ = 0.9823 R^2^_Adj_ = 0.9586 Signal-to-noise ratio (S/N = 13.8982)						

“*” indicates a significant impact on the results (*p* < 0.05); “**” indicates an extremely significant impact on the results (*p* < 0.01).

Using the experimental data, Design-Expert 3.7 was utilized to perform multiple regression fitting. A quadratic multiple regression equation was established to describe the relationship between the influencing factors—enzyme addition amount, inoculation amount, time, temperature, and the GI value. The general form of the regression equation is:

Y = 48.71–0.1242 × A-0.3883 × B-0.1533 × C + 0.2075 × D-0.1925 × A × B-0.0475 × A × C + 0.1375 × A × D-0.0175 × B × C-0.2200 × B × D + 0.2350 × C × D + 1.31 × A2 + 0.3390 × B2 + 0.7715 × C2 + 0.4752 × D2.

where Y represents the GI value, A represents the amount of enzyme added, B represents the number of bacteria inoculated, C represents the time, and D represents the temperature.

With GI as the response variable, the quadratic regression model was extremely significant (*p* < 0.01) (Table 5). The lack-of-fit terms of the regression equation showed no statistical significance (*p* = 0.0781), indicating that unknown factors had minimal interference with the experimental results, as *p* > 0.05. The coefficient of determination (*R*^2^ = 0.9823) and the adjustment coefficient of determination R^2^_Adj_ = 0.9586 indicate that the GI value of corn flour processed by the co-treatment with bacteria and enzymes primarily depends on the amount of enzyme dosage, inoculation amount, fermentation time, and fermentation temperature. The signal-to-noise ratio (S/N) = 13.8982 > 4 also indirectly confirms that this model has a certain degree of credibility.

**Figure 4:**
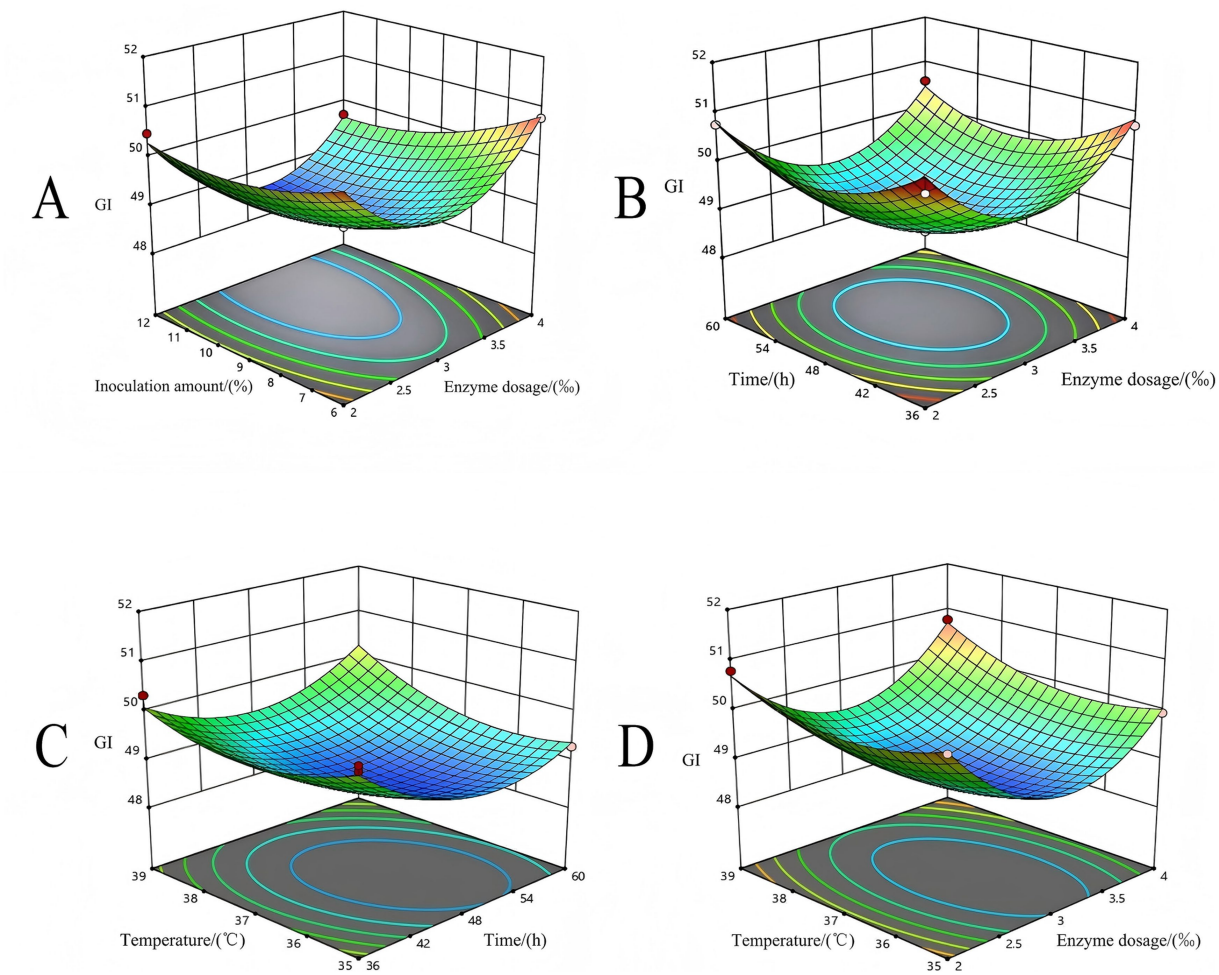
Optimization of fermentation process using Box–Behnken experimental design. **(A)** Surface diagram of the influence of enzyme dosage and bacterial dosage on GI value. **(B)** Surface diagram of the influence of enzyme dosage and time on GI value. **(C)** Surface diagram of the influence of time and temperature on GI value. **(D)** Surface diagram of the influence of enzyme dosage and temperature on GI value.

Through the analysis of variance of the regression equation, the influence degrees of the four factors on the GI value were B > D > C > A, that is, the inoculation amount > temperature > time > enzyme addition amount. Among these, factor B (inoculation amount) had an extremely significant impact on the result (*p* < 0.01), while factors C (time) and D (temperature) had significant impacts on the result (*p* < 0.05). Factor A (enzyme dosage) showed no significant impact on the result (*p* > 0.05). Interaction terms such as AB, AC, AD, BC, BD, and CD had no significant influence on the results (*p* > 0.05), whereas all quadratic terms had an extremely significant impact on the results (*p* < 0.01). The 3D curve of the response surface of the interaction of enzyme dosage, inoculation amount, temperature, and time on the GI value, as determined by the regression equation, is shown in Figure 4.

At a fixed time of 48 h and temperature of 37 °C, the GI value shows a trend of first decreasing and then increasing with the increase in the inoculation amount and the enzyme dosages (Figure 4A). When the amount of enzyme added is 3‰ (90 U/g), the GI value gradually decreases as the inoculation amount increases. The lowest GI value was observed when the enzyme dosage was 2.581‰ (77.43 U/g), and the inoculation amount was 1.03 × 10^8^ CFU/mL. The elliptical contour lines and concave response surface indicate that the interaction between these two factors was statistically significant and yielded a clear minimum value.

As shown in Figure 4B, the value of GI decreased with the increase in fermentation time at a fixed inoculum size of 9 × 10^7^ CFU/mL, temperature of 37 °C, and the enzyme dosage of 3‰, but began to increase after reaching the threshold. This might be due to the prolonged fermentation, leading to the depletion of nutrients in the substrate, which was not conducive to bacterial growth and metabolic activities. The interaction between enzyme dosage and fermentation time was significant, and the lowest GI value was observed at an enzyme dosage of 2.581‰ and a fermentation time of 49.251 h, representing the valley point of the response surface.

Figure 4C shows that when the inoculum size was fixed at 9 × 10^7^ CFU/mL, and the enzyme dosage was 3‰, the GI value showed a trend of first decreasing and then increasing with an increase in temperature and time. At a fixed temperature of 37 °C, the GI value gradually decreased with an increase in time. The lowest GI value was obtained when the time was 49.25, and the temperature was 36.83 °C. The inclination angle of the time surface was smaller than that of the temperature surface. Therefore, it is inferred that the influence of temperature on the results is greater than that of time.

At a fixed inoculation size of 9 × 10^7^ CFU/mL and fermentation time of 48 h, the GI value exhibited a trend of first decreasing and then increasing with the increase of enzyme dosages and temperature, as shown in Figure 4D. The lowest GI value was observed when the enzyme dosage was 2.58‰, and the temperature was 36.83 °C, representing the valley point of the response surface. The elliptical contour map and the presence of concave points suggest that there is the strongest interaction between enzyme dosage and temperature among the tested factor combinations, exerting a significant influence on GI reduction.

The optimal fermentation process conditions obtained by the response surface experiment were: enzyme dosage of 2.581‰ (77.43 U/g), fermentation time of 49.25 h, inoculation amount of 1.03 × 10^8^ CFU/mL, and temperature of 36.831 °C. Under these conditions, the predicted value of GI was 48.91. Fermentation was carried out under these conditions, and 3 to 5 groups of parallel experiments were conducted. The final result was 48.87, which was close enough to the model prediction value. Therefore, the regression model can effectively reduce the GI value. Therefore, the co-fermentation of bacteria and enzymes adopted in this study is a comprehensive bioprocessing strategy. Beyond achieving GI reduction, this method is more likely to simultaneously improve the flavor and nutritional quality of the product, offering a dual benefit compared to single-mechanism interventions.

### 3.5 Determination of the water-holding capacity and gel strength of corn flour

**Figure 5:**
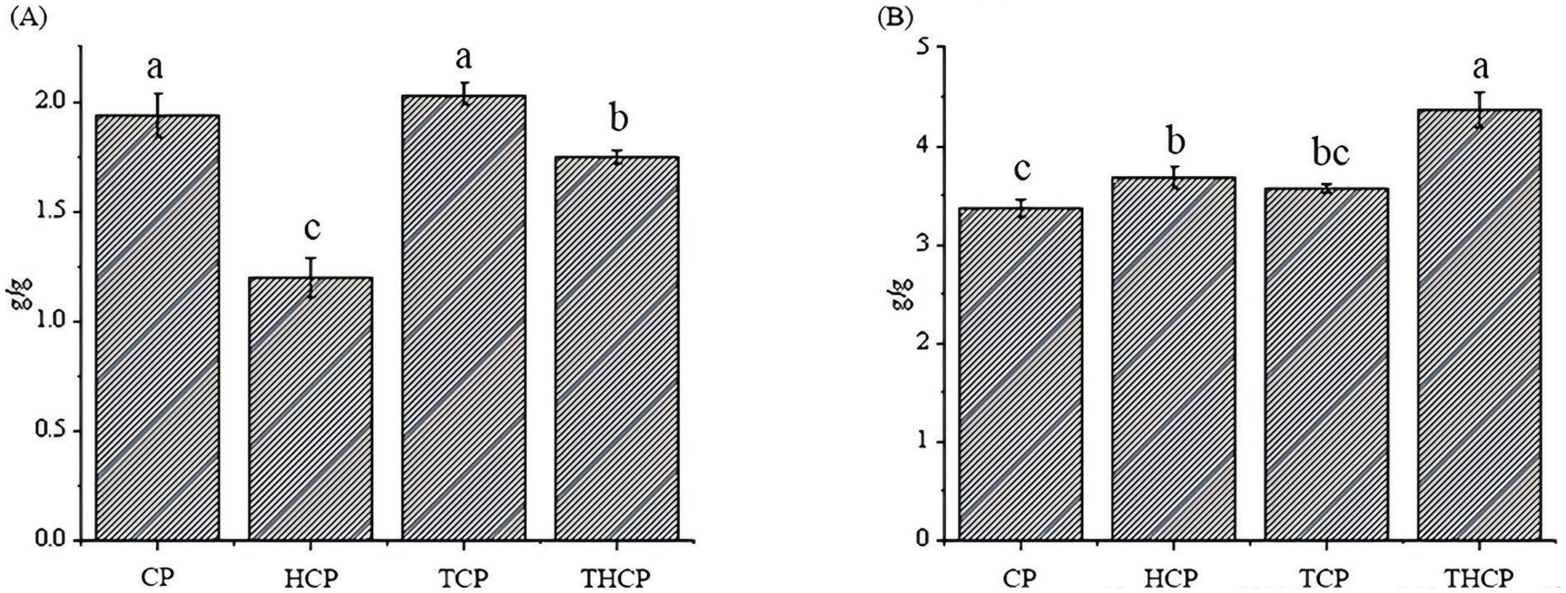
Measurement of water-holding capacity and gelation capacity of corn flour. **(A)** Effect of different treatment conditions on the water-holding capacity of corn flour. **(B)** Effect of different treatment conditions on the gelation capacity of corn flour.

The effects of various treatments on the functional properties and macroscopic structure of corn flour are comprehensively shown in Figure 5 (CP: control; HCP: *Lactobacillus HR* fermentation; TCP: TGase treatment; THCP: *Lactobacillus HR* combined with TGase). As shown in Figure 5, the water-holding capacity of corn flour treated by the synergy of *Lactobacillus HR* fermentation and TGase modification decreased by 10.3% (*p* < 0.05), and the gelation force increased by 29.7% (*p* < 0.05). Notably, the TCP group exhibited the highest water-holding capacity, significantly surpassing all other groups. This was followed by the control group (CP), the THCP group, and finally the HCP group (Figure 5A). Regarding gel strength, THCP showed the highest value, which was significantly greater than that of TCP, and both enzyme-treated groups outperformed the singly *Lactobacillus HR* fermented (HCP) and control (CP) samples (Figure 5B).

The change in water-holding capacity and gel properties can be attributed to the protein cross-linking action of TGase, which promotes the formation of a robust three-dimensional network. *Lactobacillus HR* fermentation may further contribute by moderately hydrolyzing proteins and exposing additional cross-linking sites. Under the combined action of *Lactobacillus HR* and TGase, the network structure is optimized, leading to the greatest improvement in gel performance. These results clarify the role of TGase in strengthening the water retention and gelling properties of protein matrices and highlight the synergistic potential of combined enzymatic modification.

### 3.6 Changes in the nutritional composition of corn flour

As summarized in Table 6, regarding mineral content, the ash values remained highly stable across all treatment groups, with a narrow range from 1.09 ± 0.31% to 1.58 ± 0.12%. This consistency suggests that neither bacterial fermentation, TGase enzymatic modification, nor their combined application had a significant impact on the mineral retention capacity of the corn flour. In contrast, the protein content indicated substantial improvements through processing interventions. Both TGase-modified corn flour (5.59 ± 0.53%) and bacteria-enzyme co-processed corn flour (5.51 ± 0.84%) exhibited statistically higher crude protein levels compared to the control group (CP) (4.53 ± 0.89%).

**Table 6:** Nutritional composition of different types of corn meal samples.

Types of cornmeal	Ash content %	Crude protein %	Starch %	Dietary fiber %	Total sugar %
CP	1.578 ± 0.12	4.532 ± 0.89	78.048 ± 2.51^a^	5.036 ± 0.76	25.16 ± 0.09
HCP	1.323 ± 0.28	4.834 ± 0.66	69.689 ± 2.38^b^	6.886 ± 0.92	24.32 ± 0.02
TCP	1.376 ± 0.14	5.591 ± 0.53	71.908 ± 2.73^ab^	6.764 ± 0.83	24.78 ± 0.07
THCP	1.093 ± 0.31	5.507 ± 0.84	65.193 ± 1.99^b^	7.977 ± 1.25	23.40 ± 0.04

Data represent mean ± SD of triplicate measurements. Superscript letters (a, b) denote statistically significant differences (*p* < 0.05) within each parameter. CP, control; HCP, *Lactobacillus HR* fermentation; TCP, TGase treatment; THCP, *Lactobacillus HR* combined with TGase.

The starch composition analysis yielded notably intriguing findings. The CP group possessed the highest starch content (78.05 ± 2.51%), while the TGase-modified group showed a significant reduction to 71.91 ± 2.73% (*p* < 0.05). In contrast, the HCP (69.69 ± 2.38%) and THCP (65.19 ± 1.99%) groups demonstrated the lowest starch concentrations. This significant consumption reveals that *Lactobacillus HR* and enzymatic treatment preferentially utilize readily hydrolysable starch fractions as carbon sources. Correspondingly, dietary fiber content showed the most significant improvement in the THCP group (7.98 ± 1.25%), representing a 60% increase compared to the CP group (5.04 ± 0.76%).

Total sugar analysis revealed consistent reductions across all processed samples, with the bacteria-enzyme co-modification group achieving the lowest level at 23.40 ± 0.04% compared to the CP group (25.16 ± 0.09%).

The above results clearly underscore the nutritional advantages of co-processing corn flour with bacteria and enzymes. The observed reduction in total sugar content effectively lowers the product’s glycemic index (GI). This is achieved not only through the direct delay in glucose release due to a decrease in available sugar content, but also via the elevated crude protein levels, which might physically encapsulate starch granules and interact with them, thereby impeding *α*-amylase hydrolysis to some extent and further slowing carbohydrate digestion and absorption.

### 3.7 Observation of the surface microstructure of corn flour

**Figure 6:**
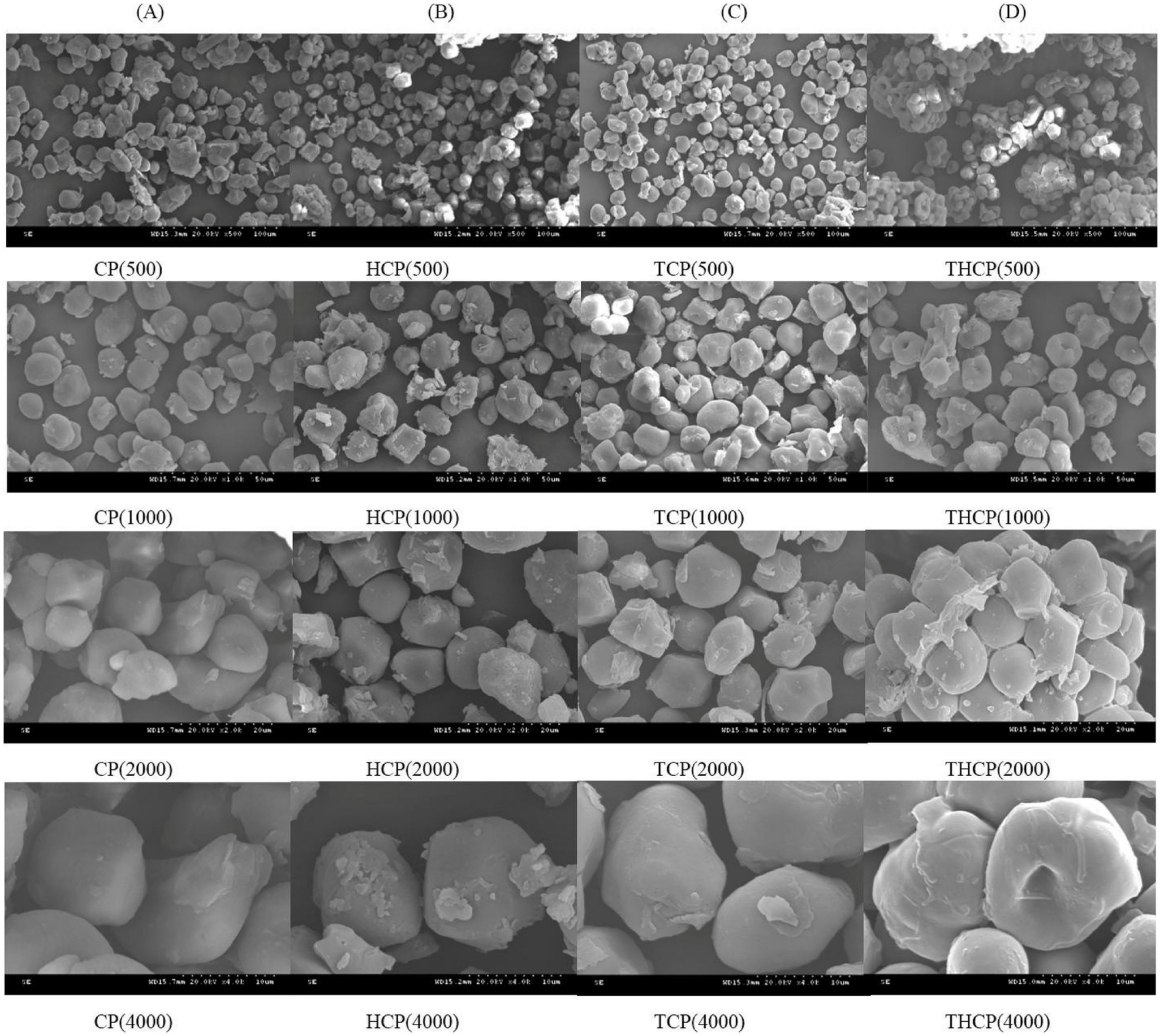
Effect of different treatment methods on the morphology of corn meal **(A–D)**. CP, Corn meal; HCP, Heated corn meal; TCP, Traditional corn meal; THCP, Traditional heated corn meal.

The microscopic morphology of corn particles under magnification of 500×, 1,000×, 2,000×, and 4,000× was observed during bacteria-enzyme co-processing (Figure 6). The particle surfaces of the group CP were smooth, and the distribution was relatively uniform. The particles in group HCP were unevenly distributed, varying in shape and size. The surface was rough, with flocculent substances, depressions, protrusions, and distinct edges and corners. Under the action of *Lactobacillus HR,* extensive hydrolysis causes a reduction in particle size, producing irregular shapes and, in some cases, fragmented starch granules. TGase-modified corn flour (TCP) exhibited a rough, textured surface with depressions and flocculent substances, showing a phenomenon of particle breakage and creating irregular spherical shapes. Moreover, Figure 6D shows that after the co-treatment of *Lactobacillus HR* and TGase (THCP), starch particles aggregate and cross-link with proteins, which can effectively prevent the contact between starch molecules and hydrolases, increase the content of RS, and reduce the GI value.

### 3.8 Fourier transform infrared spectroscopic analysis

**Figure 7:**
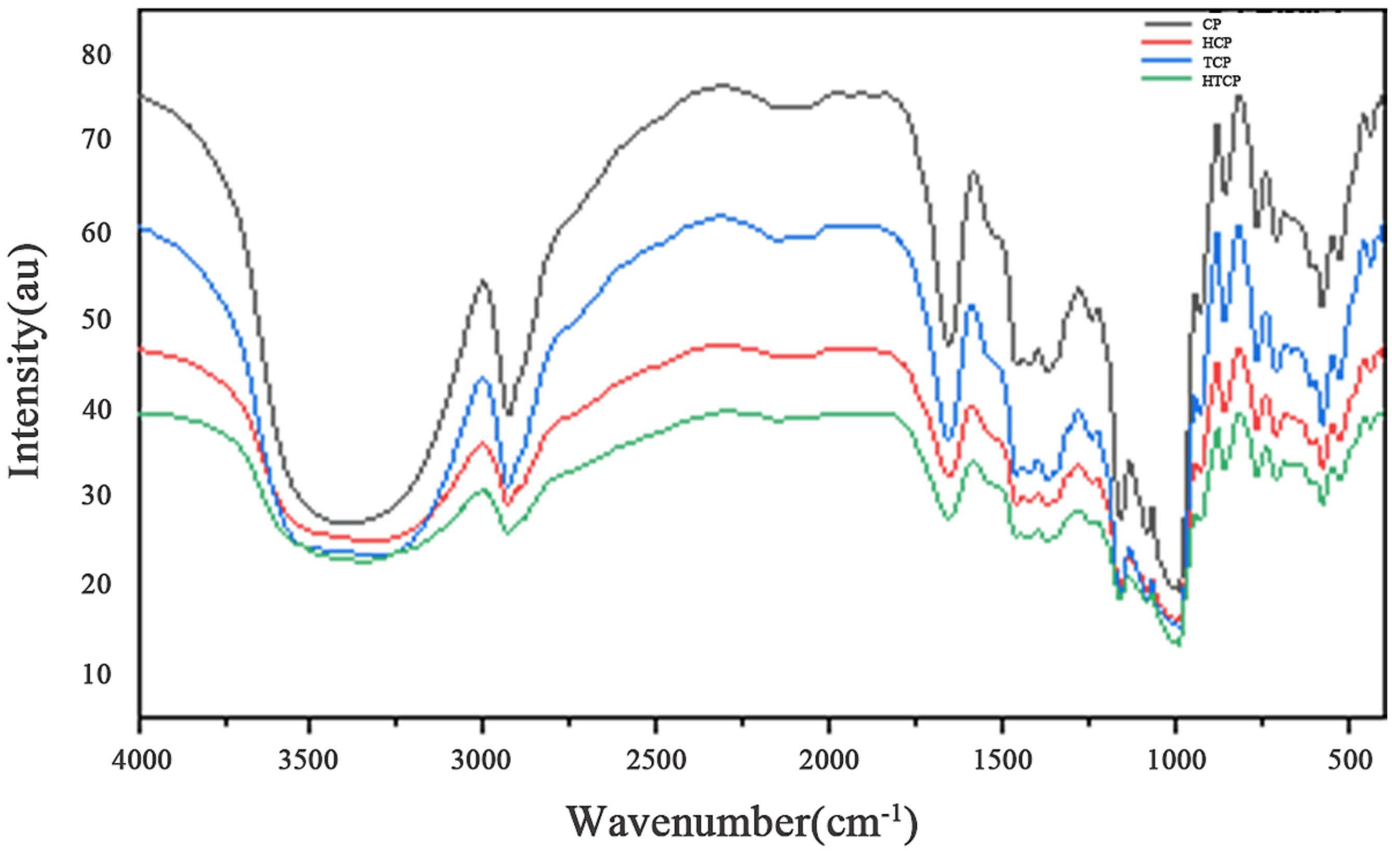
Fourier infrared spectrogram of modified corn flour.

Fourier transform infrared spectroscopy (FTIR) was utilized to analyze the structural changes of functional groups in processed corn flour (Figure 7). No new absorption peaks emerged in the corn flour after modification, indicating that no novel chemical functional groups were formed. There were some spectral changes between 500 and 1,500 cm^−1^, corresponding to elongation vibrations of C-C, C-OH, and C-H bonds in starch (39). The absorption peak at 2,929 cm^−1^ is related to the C-H stretching vibration of the system. It became flatter after treatment, indicating starch molecular chain breakage under bacterial-enzyme co-processing. Peaks near 1,047 cm^−1^ and 1,022 cm^−1^ reflected the crystalline and non-crystalline regions of starch, respectively, highlighting alterations in molecular order between ordered and disordered structures. Additionally, the absorption peak near 995 cm^−1^ is caused by the bending vibration of C-OH. Corresponding to the hydrogen bond structure formed among starch molecules, further indicating structural reorganization that may influence digestibility and glycemic response.

The ratio of the absorption peak intensities at 1,047/1,022 cm^−1^ and 1,022/995 cm^−1^ serves as an indicator of the ordered structure of starch (40). As shown in Tables 7, a larger 1,047/1,022 cm^−1^ ratio corresponds to a higher degree of molecular ordering within starch. After the co-treatment of bacteria and enzymes, the ratio of 1,047:1,022 cm^−1^ indicates an increase in the orderliness of the starch. Consequently, the structurally modified starch exhibits anti-digestive properties, reducing susceptibility to hydrolysis and thereby contributing to the observed decline in GI.

**Table 7:** FTIR spectrum (1,047/1,022) cm^−1^ ratio of different types of corn meal samples.

Types of cornmeal	(1,047/1,022) cm^−1^
CP	1.068
HCP	1.135
TCP	1.090
THCP	1.146

CP, control; HCP, Lactobacillus HR fermentation; TCP, TGase treatment; THCP, *Lactobacillus HR* combined with TGase.

### 3.9 Determination of amino acid content

As shown in Table 8, compared to the CP group, the total amino acid content showed an increase after treatment, while a small portion of the amino acid content experienced a slight decrease. Notably, the glycine (Gly) content increased significantly by 57.79% through bacterial-enzyme collaborative processing. Among the essential amino acids, particularly Lysine increased by 38.11% after fermentation, 14.23% after TGase enzymatic hydrolysis, and 12.45% after synergy. The content of Thr increased by 17.53% after fermentation, 3.94% after enzymatic hydrolysis, and 2.27% after bacterial-enzyme synergy. The changes in the contents of Tyr, Phe, Lys, and other amino acids are mainly attributed to the *Lactobacillus HR* metabolism. The hydrolysis of corn proteins by TGase releases amino acids. These amino acids are subsequently harnessed by *Lactobacillus HR* to support its metabolic activities and growth. However, the synergistic intervention of *Lactobacillus HR* and TGase still yielded a significant overall increase in TAA. The increase in lysine, methionine, and alanine contents enhances the nutritional value of the corn flour, while phenylalanine and tryptophan showed slight decreases. Therefore, the impact on the properties of the corn flour is relatively positive. The general increase in content may be attributed to the hydrolysis of proteins into smaller molecules, where peptide bonds in proteins or peptide chains are broken.

**Table 8:** Changes of amino acid content.

Name	CP	HCP	TCP	THCP
Asp	2.917 ± 0.123^a^	3.348 ± 0.141^b^	2.784 ± 0.118^a^	3.165 ± 0.132^ab^
Thr	1.854 ± 0.082^a^	2.179 ± 0.095^b^	1.927 ± 0.085^a^	1.896 ± 0.083^a^
Ser	3.022 ± 0.131^a^	3.850 ± 0.162^b^	3.117 ± 0.135^a^	3.104 ± 0.134^a^
Glu	9.203 ± 0.215^a^	9.573 ± 0.232^a^	9.659 ± 0.238^a^	9.470 ± 0.229^a^
Gly	3.016 ± 0.142^a^	3.853 ± 0.176^b^	2.973 ± 0.138^a^	4.759 ± 0.208^c^
Ala	5.557 ± 0.201^a^	6.441 ± 0.243^b^	5.686 ± 0.207^a^	6.304 ± 0.236^b^
Cys	0.230 ± 0.015^a^	0.245 ± 0.016^a^	0.249 ± 0.017^a^	0.189 ± 0.012^b^
Val	2.391 ± 0.105^b^	2.043 ± 0.092^a^	2.541 ± 0.112^b^	2.575 ± 0.115^b^
Met	0.737 ± 0.032^a^	0.851 ± 0.038^b^	0.794 ± 0.035^ab^	0.827 ± 0.037^ab^
Ile	1.441 ± 0.065^a^	2.098 ± 0.093^b^	1.548 ± 0.069^a^	1.535 ± 0.068^a^
Leu	6.647 ± 0.223^b^	6.847 ± 0.235^b^	6.942 ± 0.241^b^	6.446 ± 0.218^a^
Tyr	0.375 ± 0.021^b^	0.550 ± 0.028^c^	0.445 ± 0.023^b^	0.215 ± 0.014^a^
Phe	1.849 ± 0.086^b^	2.182 ± 0.098^c^	1.955 ± 0.089^b^	1.638 ± 0.075^a^
Lys	0.787 ± 0.035^a^	1.087 ± 0.048^b^	0.899 ± 0.039^ab^	0.885 ± 0.037^ab^
His	4.253 ± 0.186^a^	4.282 ± 0.188^a^	4.140 ± 0.181^a^	4.589 ± 0.199^b^
Arg	1.119 ± 0.052^a^	1.281 ± 0.058^b^	1.171 ± 0.054^a^	1.114 ± 0.051^a^
Pro	1.026 ± 0.047^b^	1.101 ± 0.051^b^	1.116 ± 0.052^b^	0.889 ± 0.041^a^
TAA	46.424 ± 1.089^a^	51.811 ± 1.256^c^	47.946 ± 1.103^ab^	49.600 ± 1.201^b^

Data represent mean ± SD of triplicate measurements. Superscript letters (a, b, c) denote statistically significant differences (*p* < 0.05) within each parameter. CP, control; HCP, *Lactobacillus HR* fermentation; TCP, TGase treatment; THCP, *Lactobacillus HR* combined with TGase.

The *Lactobacillus HR* initially hydrolyzes starch and protein, providing more terminal and defect sites for the starch molecular chains. Meanwhile, TGase can indirectly stabilize these starch fragments by promoting the formation of protein cross-linking networks, thereby reorganizing them into a more ordered and compact arrangement. Therefore, the resulting resistant-to-digestion starch is a molecularly ordered and structurally enhanced starch. This starch exhibits higher resistance to enzymatic hydrolysis, reduces sensitivity to enzymatic hydrolysis, and thus contributes to the observed GI reduction.

## 4 Discussion

Corn, as an important economic crop worldwide, has a relatively lower digestibility compared with other staple foods (rice and wheat), but its GI still needs to be further reduced. In this study, corn flour was first fermented with *Lactobacillus HR*, resulting in an increase in its RS content. This effect is mainly attributed to biological fermentation, which facilitates the formation of porous structures and enhances orderliness. Moreover, bacteria–enzyme synergy may degrade and recombine amylopectin, further increasing the amylose content and promoting the formation of starch–lipid/starch–protein complexes (41). Meanwhile, microbial fermentation also consumes a portion of the starch, thereby increasing the relative proportion of resistant starch in corn flour. TGase utilizes the *ε*-amino group of lysine residues as the acyl acceptor to induce intramolecular or intermolecular cross-linking reactions, which alter the texture of corn flour and impart unique textural properties and adhesive properties (42). Therefore, after the combined treatment with microorganisms and enzymes at 36.831 °C for 49.251 h, the GI value of corn flour was finally reduced to 48.87.

The microscopic structural changes led to a decrease in the glycemic index. Importantly, a synergistic association was observed between TGase and *Lactobacillus HR*. TGase-mediated protein cross-linking generated a network structure that may provide a favorable growth environment or attachment sites for *Lactobacillus HR*, thereby enhancing the fermentation efficiency of *Lactobacillus HR* and lowering the GI value. Under the influence of co-modification, protein cross-linking can increase gel strength and reflect the agglutination ability of proteins. This cross-linked protein matrix can interact with starch granules, encapsulating them to form starch–protein complexes (43). These complexes can act as a physical barrier, restricting the accessibility of starch hydrolases to starch, thereby directly promoting the formation of RS and contributing to the observed GI reduction (44). In addition, this reinforced structure may also inhibit starch expansion and dissolution during gelatinization, thereby leading to a decrease in digestion rate (45). This finding is consistent with the mechanism reported by Sun et al. (46), which demonstrated that ordered starch–protein complexes can directly delay digestion, thereby jointly promoting the formation of resistant starch (RS) and the decrease in GI.

In terms of nutritional composition, the proportion of dietary fiber has risen substantially. This increase not only indicates a reduction in starch concentration but also implies that the co-modified corn flour may regulate glycometabolism via abundant hydrolysis-resistant components. Meanwhile, dietary fiber serves as the key substrate for colonic microbiota fermentation, supporting gut health and microbial balance (47). The stable ash content ensures the preservation of essential mineral elements (41). Moreover, the total amino acid content (TAA) of the processed corn flour has significantly increased, enhancing its nutritional value. These high-level amino acids play a crucial role in regulating glucose metabolism throughout the body (48). For instance, the increase in alanine content plays a key role by transporting amino groups between the liver and muscles through the glucose–alanine cycle, regulating systemic glucose metabolism, and activating AMP-activated protein kinase (AMPK) in the liver to promote glucose uptake and fatty acid oxidation, thereby improving energy metabolism (49). Therefore, beyond its low glycemic index (GI) property, corn flour treated through *Lactobacillus HR*-TGase synergy is enriched with amino acids that can modulate glucose and lipid metabolism pathways, thereby influencing glucose uptake and metabolic regulation in the human body.

In conclusion, the combined enzymatic and microbial modification not only modifies starch, but also prevents enzymatic hydrolysis by constructing a protective protein matrix, thereby reducing the GI. This strategy simultaneously achieves the comprehensive goals of low GI, enhanced functional properties, and optimized nutritional quality. It provides strategies for the deep processing of corn flour, possessing significant academic value and possessing broad prospects for industrial applications. However, this *in vitro* study was conducted only on corn flour and lacked in-depth analysis at the mechanism level. Therefore, in the future, it is necessary to conduct *in vivo* verification to further confirm its effectiveness, and to utilize multi-omics methods to explore the synergistic effects between microorganisms, to apply it to other starch-containing substrates.

## 5 Conclusion

Owing to its high starch content, corn flour serves as an effective substrate for the preparation of low-GI products through the synergistic action of the *Lactobacillus HR* and TGase. Single-factor experiments combined with response surface optimization methodology identified the optimal conditions as an enzyme dosage of 2.581‰ (77.43 U/g), treatment time of 49.251 h, inoculation amount of 1.03 × 10^8^ CFU/mL, and temperature of 36.831 °C, yielding a GI value of 48.87, soluble protein content of 5.96 mg/g, and resistant starch content of 51.19%. FTIR analysis revealed that no new chemical functional groups were formed, while the increased 1,047/1,022 cm^−1^ ratio indicated enhanced starch orderliness, which in turn improved anti-digestive properties. Microscopic observations further confirmed the porous structures in co-modified starch, indicating an increase in the amylose content and higher resistant starch levels, thereby contributing to GI reduction. Collectively, these findings demonstrate that bacteria–enzyme co-processing can produce nutritionally enhanced, low-GI corn flour, offering an innovative pathway for the development of healthy staple food. Future work should focus on applying this process to other cereal grains and validating its *in vivo* efficacy to fully realize its application potential.

## Data Availability

The original contributions presented in the study are included in the article/supplementary material, further inquiries can be directed to the corresponding authors.
